# Effective e-learning in surgical education: the core values underpinning effective e-learning environments and how these may be enhanced for future surgical education

**DOI:** 10.3332/ecancer.2016.ed53

**Published:** 2016-02-16

**Authors:** R Bamford, J Coulston

**Affiliations:** Health Education South West, Severn Postgraduate School of Surgery, Deanery House, Unit D-Vantage Business Park, Old Gloucester Road, Bristol, BS16 1GW, UK

## Abstract

e-learning is a valuable tool that has a number of advantages for Surgical Oncology training and education. The rapidly evolving nature of, and limited clinical exposure to oncological practice creates challenges for surgical trainees to stay up to date and engaged. Online learning can be accessed anywhere at any time and allows trainees to develop, apply and be assessed on their learning. To be effective, it must be educationally sound and embrace technology to enhance learners’ experience.

## Introduction

Modern technology is rapidly advancing and allows for an equally increasing vast array of opportunities in medical education. The vast majority of NHS trusts now offer online learning tools and modules for continued professional development. Within Surgical Training, the Royal College of Surgeons of England support online learning modules and the Royal College of Surgeons of Ireland mandate their surgical trainees to use their online learning management system [1, 2]. The UK medical regulatory body, the GMC, and the UK Department of Health support the use of e-learning as part of blended learning process for all NHS staff [3, 4].

## Benefits of online e-learning

The benefits of online e-learning are most often described in the operational and logistical benefits that e-learning can offer. The Joint Information Council offer 11 advantages to e-learning [5]. The majority of these points confer on e-learning being accessible and flexible to learners. This is because online e-learning can be made available on a range of platforms and accessible at work or home. This flexibility allows trainees to access their learning environment at a time that is convenient and relevant to their own training needs or as part of a blended approach with more traditional teaching. Online e-learning can take many different forms that can be adapted to suit the need for specific learning environments and learners. These include web-based data resources [6], interactive online modules [7], virtual reality environments and virtual patients. Often, individuals or institutions pull these resources together as part of an online learning platform [8]. In doing so, online e-learning has developed to be the process of learning that is supported with information and communications technology.

The impact of the European Working Time Directive, the increasing workload of trainees, reduced study budgets and constantly updating evidence of best practise signal that these advantages are hugely relevant to Surgical Trainees and Continued Professional Development. This is especially true within oncology and oncological surgery where the development of new treatments and treatment pathways are constantly evolving. E-learning allows for new evidence and pathways to be seen by a trainee and also allows them to apply and be assessed on the new information in a safe setting. This far outweighs the inevitable delay in printed evidence and textbooks. Furthermore, the use of online interactive technology such as forums or social media allow for such advances to be discussed and debated across the medical landscape and around the world. This exchange of ideas and discussion can generate a wealth of valuable information and learning opportunities for the user.

The development of interactive, open access organ specific cancer modules for surgical trainees allow for the user to access information that they would otherwise have limited exposure to [9]. In this setting, trainees can work through all aspects of oncological disease from presentation, investigation, oncological and surgical treatment plans and be directly involved in the role of other healthcare specialists such as Palliative Care or Radiology.

As technology improves the opportunities for e-learning and the perceived benefits will undoubtedly increase, however, it is essential that e-learning also encompass high educational standards that ensure the greatest benefit to the learners.

## Educational standards

Surgical training requires the development of knowledge, technical skills, non-technical skills and attitudes. It is important that e-learning is used to develop the appropriate skills set in each of these and the appropriate tool is used for each. Bloom’s taxonomy of skills, knowledge and attitudes is often quoted within medical education. E-learning tools are usually placed within the knowledge domain and have the opportunity to offer up to date information readily.

According to Kolb, learning is an active process through a cycle of experience, reflection, conceptualisation and experimentation [10]. In the US, The National Training Laboratories support this concept and identify increased retention of knowledge in students who are increasingly active and challenged in their learning [11]. If trainees can be exposed to interactive and multi-sensory experiences then their level of recall can be significantly increased from that of listening or reading in a traditional educational model. E-learning offers the opportunity to achieve this and should be considered when embarking on an e-learning design.

If used appropriately, e-learning also facilitates the development of knowledge towards the pinnacle of Bloom’s taxonomy of knowledge and allows a learner not only to recall the information but also to comprehend and apply it to a clinical situation. As technology improves, there are greater opportunities to synthesise this information and make decisions and judgements based on the information available. This decision making process can also be applied within the attitude domain of learning and allows for a safe environment in which novices can develop into competent trainees with skills relevant to clinical practice.

All of these methods are wasted if the content of the module is not relevant. In post-graduate surgical education it is vital that the best available, up to date and critically appraised evidence supports the information contained within the modules. Furthermore, for trainees to find the modules relevant, the content should be linked to their curriculum and ideally supported by the training body leading their progression.

## Developing high quality e-learning resources

Creating an effective Surgical e-learning environment can be time consuming and expensive. Not only does it require an understanding of educational principles and the ability to critically appraise and assimilate the required information, it also requires creativity and a willingness to experiment with new ideas and concepts. Often, it is the end user who has the most interest in ensuring that these programmes are of high quality and are, therefore, a valuable and motivated resource that should be harnessed. These trainees often arrive with a vast understanding of technology and a willingness to engage in the process to see how much can be possible. To achieve the high fidelity and standard that is required in a finished product, collaboration with professional companies can also be hugely beneficial.

Ensuring the quality of a resource’s contents and design requires a robust system of peer review and testing. Chosen peer reviewers should be able to comment on the content and accuracy of the resource and to ensure the use of educational principles. A rigorous piloting process should be put in place to ensure the usability is appropriate and the desired learning objectives can be met.

## The future of surgical e-learning

Advances in technology allow for e-learning programmes to be highly interactive and immersive, whether that involves interactive models or question and answer designs in a knowledge acquisition process or the more advanced virtual patient model. In the latter design, trainees come face to face with an interactive clinical setting and progress through scenarios making the relevant clinical decisions and choices as they go. Importantly, trainees, and their virtual patient, have to live (or not) with their decisions and receive feedback as to the choices they have made. This safe environment allows for mistakes to be made as a trainee experiments with their understanding of the knowledge and available evidence on a subject whilst being highly clinically relevant and realistic. These interactive models can be created simply with text on the page or with high fidelity avatars that communicate directly with the learner. Virtual reality technology is also advancing rapidly and more affordable and realistic virtual reality environments are becoming available. These will eventually allow a trainee to walk into a clinic, emergency department, ward or operating theatre and make decisions and perform relevant tasks. Whilst these technological advances are exciting they must not be the driving force for the development of these tools. Rather, the educational need and critical evidence of their success should drive their development and introduction to surgical training as part of a blended learning programme.

The future of surgical e-learning is encouraging and does not need to occur in isolation. Effective e-learning modules can be used as a complementary tool to traditional learning methods. Ensuring a learner has completed a required learning module before attending a face-to-face training event allows for a baseline level to be reached and facilitates a meaningful and more analytical discussion of the subject.

## Conclusion

High educational standards with relevant content are essential to ensure that e-learning is a valuable tool for Surgical Education. Motivated, collaborative teams have the greatest opportunity to utilise the available technology and develop highly interactive and immersive environments that can complement surgical training.

## Competing interests

None declared.

## Funding

None declared.

## Ethical approval

Not required.
